# The Identikit of Patient at Risk for Severe COVID-19 and Death: The Dysregulation of Renin-Angiotensin System as the Common Theme

**DOI:** 10.3390/jcm10245883

**Published:** 2021-12-15

**Authors:** Riccardo Sarzani, Massimiliano Allevi, Federico Giulietti, Chiara Di Pentima, Serena Re, Piero Giordano, Francesco Spannella

**Affiliations:** 1 Internal Medicine and Geriatrics, IRCCS INRCA, Via della Montagnola 81, 60127 Ancona, Italy; m.allevi@inrca.it (M.A.); f.giulietti@inrca.it (F.G.); c.dipentima@inrca.it (C.D.P.); s.re@inrca.it (S.R.); p.giordano@inrca.it (P.G.); f.spannella@univpm.it (F.S.); 2 Department of Clinical and Molecular Sciences, University “Politecnica delle Marche”, Via Tronto 10/a, 60126 Ancona, Italy

**Keywords:** COVID-19, SARS-CoV-2, cardiovascular disease, renin-angiotensin system, older age, overweight, obesity, hypertension, metabolic syndrome, diabetes mellitus

## Abstract

Since the first months of the coronavirus disease 2019 (COVID-19) pandemic, several specific physiologic traits, such as male sex and older age, or health conditions, such as overweight/obesity, arterial hypertension, metabolic syndrome, and type 2 diabetes mellitus, have been found to be highly prevalent and associated with increased risk of adverse outcomes in hospitalized patients. All these cardiovascular morbidities are widespread in the population and often coexist, thus identifying a common patient phenotype, characterized by a hyper-activation of the “classic” renin-angiotensin system (RAS) and mediated by the binding of angiotensin II (Ang II) to the type 1-receptor. At the same time, the RAS imbalance was proved to be crucial in the genesis of lung injury after severe acute respiratory syndrome coronavirus 2 (SARS-CoV-2) infection, where angiotensin-converting-enzyme-2 (ACE2) is not only the receptor for SARS-CoV-2, but its down-regulation through internalization and shedding, caused by the virus binding, leads to a further dysregulation of RAS by reducing angiotensin 1-7 (Ang 1-7) production. This focused narrative review will discuss the main available evidence on the role played by cardiovascular and metabolic conditions in severe COVID-19, providing a possible pathophysiological link based on the disequilibrium between the two opposite arms of RAS.

## 1. Introduction

The coronavirus disease 2019 (COVID-19), caused by the severe acute respiratory syndrome coronavirus 2 (SARS-CoV-2), was first reported in December 2019 in Wuhan, China [1]. Since then, COVID-19 has exponentially spread all around the world, making the World Health Organization (WHO) declaring it a pandemic on March 11, 2020. Until now, there have been millions of deaths worldwide. Since the first months of the pandemic, several specific physiologic traits, such as male sex and older age, or health conditions, such as overweight/obesity, arterial hypertension, metabolic syndrome, and type 2 diabetes mellitus, have been found to be highly prevalent and associated with increased risk of adverse outcomes in hospitalized COVID-19 patients [2]. The first report on hospitalized COVID-19 patients in Wuhan found that hypertension (31.2%), diabetes mellitus (10.1%), cardiovascular disease (14.5%), and malignancy (17.2%) were the most common related health conditions [3]. In March 2020, the American College of Cardiology (ACC) issued a clinical bulletin confirming that patients suffering from hypertension, diabetes, and cardiovascular disease had higher case fatality rates than the average population. In a large case series of sequentially hospitalized COVID-19 patients in the New York City area between March 2020 and April 2020, the most common comorbidities were hypertension (56.6%), obesity (41.7%), and diabetes (33.8%) [4]. Furthermore, meta-analytic data confirmed significant positive correlations between COVID-19 severity and hypertension, diabetes, and coronary heart disease [5]. Case-control studies have reported that obesity, together with diabetes and hypertension, were the strongest predictors for COVID-19, being associated not only with illness severity but also with higher risk of acquiring the infection [6]. 

Why do these conditions characterize the most severe COVID-19? It is very likely that the “classic” renin-angiotensin-system (RAS) and angiotensin-converting-enzyme (ACE) 2 play a key role in this scenario. ACE2 is the cellular receptor for SARS-CoV-2 [7], but its functional down-regulation, through internalization and shedding caused by the virus binding, can lead to an imbalance between the two arms of RAS, leading to tissue damage after SARS-CoV-2 infection by unopposed angiotensin II (Ang II)-AT1 receptor (AT1R) activity [8]. A very large quantity of experimental evidence confirmed by high-quality clinical research performed before the SARS-CoV-2 pandemic demonstrated that RAS dysregulation, resulting from the disequilibrium between these two opposite arms, driven by ACE and ACE2, may be a crucial factor in the genesis of lung injury in SARS-CoV-2 infection [9]. In the following focused narrative review, we will discuss the role played by cardiovascular and metabolic conditions in determining the risk for severe COVID-19, proposing a conceivable pathophysiological link underpinning all, based on the dysregulation of RAS.

## 2. Disequilibrium between ACE and ACE2 Activity as a Potential Causal Mechanism for Severe COVID-19

The RAS plays a fundamental role in the regulation of fluid volume and blood pressure, but it also exerts a large spectrum of effects on several tissues, especially those that are microvasculature-rich, such as the lungs. Renin, a protease produced by renal juxtaglomerular cells in its active form, cleaves angiotensinogen, released mainly by the liver, to form Ang I. A pathophysiological mechanism underlying the cardio-metabolic conditions associated with a greater risk of morbidity and mortality in COVID-19 is the imbalance between ACE and its homologous ACE2, with a decreased activity of the latter and impairment of its protective effects. Similarly to ACE, ACE2 is a ubiquitous enzyme, particularly expressed in the lungs but also present on the enterocytes of the small intestine and on the endothelial cells of several organs and systems, such as the brain, cardiovascular system, and kidney [10,11]. ACE and ACE2 are key enzymes in the metabolism of Ang I: ACE, expressed widespread by endothelial cells, catalyzes the conversion of Ang I to the octapeptide Ang II [12], which exerts its effects via Ang II type 1 and type 2 receptors (AT1R and AT2R, respectively). In particular, binding of Ang II to AT1R is the one most commonly expressed in normal adult tissues, stimulating vasoconstriction, sodium reabsorption, and blood pressure increase, promoting vascular damage, inflammation, and fibrosis [13]. On the opposite side, ACE2 is a type I transmembrane metallocarboxypeptidase that cleaves Ang I into a nonapeptide [Ang (1-9)] that binds AT2R and Ang II into a heptapeptide [Ang (1-7)] that binds its specific receptor, initially identified as an “oncogene” (Mas receptor, MasR) [14]. The two main effects of ACE2 are thereby the degradation of Ang II, the principal effector of the “classic” RAS arm through AT1R, and the production of Ang (1-7), which exerts opposite effects by inducing vasodilatation as well as anti-inflammatory and anti-fibrotic pathways through binding to the MasR [15]. ACE2 also interacts with another sub-branch of RAS based on Ang peptides in which the aminoterminal aspartate is replaced by alanine (Alatensins), leading to the production of Ala-Ang (1-7) (Alamandine) that has been found to bind Mas-related G protein-coupled receptor D (MrgD) and may also protect against lung injury and fibrosis, improving vascular/endothelial dysfunction [11]. The ACE2/Ang (1-7)/MasR axis has been found to attenuate inflammation and fibrosis in experimental models to prevent heart failure and coronary heart disease [16], as well as lung injury [17,18]. Therefore, ACE2 plays a pivotal role in the modulation of the two main arms of RAS: the ACE/Ang II/AT1R axis (“classic RAS”) and the ACE2/Ang (1-7)/MasR axis (“anti-RAS”). Indeed, ACE2 antagonizes “classic RAS”, playing an essential counter-regulatory role in the activation of the “anti-RAS” [19]. As SARS-CoV-2 uses ACE2 as a primary receptor to gain entry into human cells, causing its functional down-regulation through internalization and shedding [20,21], coronavirus infection leads to a RAS dysregulation, enhancing the ACE/Ang II/AT1R pathway up to vascular “toxicity”, causing microvascular damage and dysregulated vascular permeability. This results in capillary leakage of protein and fibrin-rich edema filling alveolar spaces, also promoting oxidative stress and inflammation, leading to acute respiratory distress syndrome (ARDS) [9]. Moreover, the ACE/Ang II/AT1R axis is likely to promote the production of inflammatory cytokines, accelerate apoptosis in alveolar epithelial cells and promote extracellular matrix synthesis, resulting in lung fibrosis, a hallmark of tissue injury in SARS-CoV-2-related pneumonia [22]. On the other hand, Ang (1-7) has been found to mitigate inflammation, counteract lung fibrosis, and improve oxygenation in acute lung injury, acting as a protective factor against ARDS [17]. The main cardio-metabolic conditions associated with a worse outcome in COVID-19 are intertwined via RAS imbalance at baseline. We will discuss these conditions one by one in the following sections, assuming a key role of the disequilibrium between “classic” RAS and “anti-RAS” as common denominators in the development of severe COVID-19. 

## 3. Overweight, Obesity, Visceral Adiposity and Metabolic Syndrome

Several studies have focused on the association between obesity and adverse outcomes in patients hospitalized for COVID-19. A first meta-analysis [23] analyzed five different cohorts between January and May 2020, finding how patients with higher body mass index (BMI) had a greater risk for intensive care unit (ICU) admission and for invasive mechanical ventilation. Further meta-analyses have corroborated these findings [24,25,26]. A retrospective cohort study reported that the proportion of patients who required invasive mechanical ventilation increased according to BMI, and it was greatest in patients with BMI ≥ 35 kg/m^2^ [27]. A recent large prospective, community-based, cohort study on patients from over 1500 English general practitioners found J-shaped associations between BMI and hospital admission or death due to COVID-19 and a linear association between BMI and ICU admission [28]. The authors reported that each excess BMI unit above a BMI of 23 kg/m^2^ was associated with progressively increased hazard ratio of adverse COVID-19 outcomes (hospital admission, ICU admission, death). Interestingly, this association was amplified for people of black ethnicity compared with those of white ethnicity and for younger people (aged 20–39 years) compared with older people (aged ≥80 years) [28]. In addition to data concerning obesity, fat deposition in the abdominal region (visceral fat) and in ectopic sites such as liver, epicardium, and skeletal muscle, was identified as independent risk factor for worse severity of COVID-19. Indeed, higher visceral fat has been reported to be associated with an increased need of intensive care in both subjects older than 65 years and males [29]. Furthermore, the risk due to obesity in COVID-19 has been found to be significantly greater in obese patients with metabolic associated fatty liver disease (MAFLD) [30]. The visceral fat is an important component of metabolic syndrome (MetS), another condition associated with poor prognosis in COVID-19. In previous case series, the prevalence of MetS in hospitalized COVID-19 patients was up to 81%, with a five-fold greater risk of disease deterioration and increased mortality risk as the MetS components count increased [31,32]. 

Obesity and overweight with visceral adiposity promote increased circulating levels of Ang I, leading to an overproduction of Ang II and hyper-activation of “classic RAS” [33,34]. Adipocytes produce and release all the components of “classic RAS”, including angiotensinogen, ACE, and Ang II, with the only exception of renin, even if they express the renin receptor [35,36]. Thus, in the context of the aforementioned SARS-CoV-2-induced ACE2 downregulation, it is likely that the increased levels of both Ang I and Ang II coming from visceral adipose tissue lead to an ACE/Ang II/AT1R “storm” affecting the pulmonary microcirculation in obese COVID-19 patients [37]. In addition, obese subjects show high circulating levels of microRNAs involved in the downregulation of ACE2, resulting in further basal dysfunction of ACE2 compared to healthy subjects [38]. Adipose tissue is also involved in cytokine and adipokine secretion, contributing to a pro-inflammatory environment. Indeed, leptin, one of the main adipokines secreted by adipocytes, has been found to be related to increased Ang II levels and decreased ACE2 expression [39]. Moreover, the deficiency of the natriuretic peptide (NP) system, found in obese subjects, mainly due to an increased expression of the clearance NP receptor C [40], could further contribute to the RAS imbalance in this population. In fact, both A-type (ANP) and B-type (BNP) NPs counteract the typical features of the ACE/Ang II/AT1R axis hyper-activation (endothelial dysfunction and increased permeability, pro-inflammatory, pro-hypertrophic, and pro-fibrotic activity), while experimental studies found that ANP can prevent the reduction in ACE2 mediated by Ang II and, conversely, Ang (1-7) can increase ANP release [34,41]. These interactions with RAS, together with their natriuretic and cardio-protective effects against acute cardiac dysfunction that may develop during SARS-CoV-2 infection, give NPs an important role in COVID-19, especially if obesity and heart failure are present [42]. 

**Figure 1:**
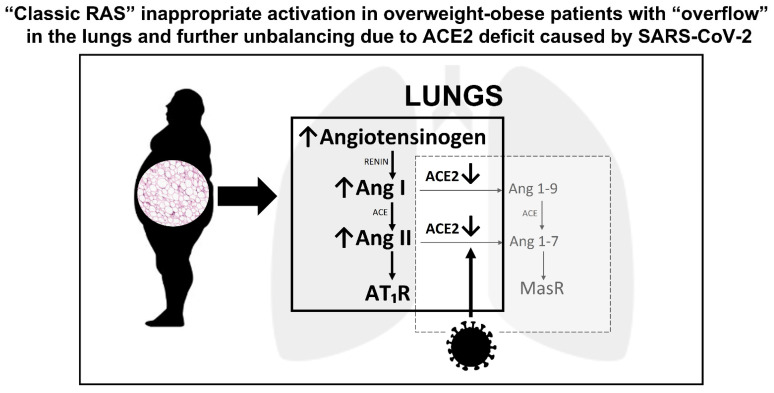
Overweight/obesity, renin-angiotensin-system, and lung injury caused by SARS-CoV-2. ACE, angiotensin-converting-enzyme; Ang, angiotensin; AT1R, angiotensin II type 1 receptor; Mas R, Mas receptor.

Overall, obesity and overweight can increase the risk for severe COVID-19 through several mechanistic, biochemical, and immunological pathways; among them, the dysregulation of RAS is likely to play a key role in increasing both disease severity and mortality (Figure 1).

## 4. Type 2 Diabetes Mellitus

Type 2 diabetes mellitus was identified early as a metabolic risk factor associated with severe COVID-19. A large meta-analysis that included 33 case-control studies, published between January and April 2020, has reported that diabetes was significantly associated with COVID-19 mortality with a pooled odds ratio of 1.90. Diabetes mellitus was also associated with severe COVID-19 with a pooled odds ratio of 2.75 [43]. The National Cohort Study in England investigated 19,256 COVID-19–related ICU admissions and reported that patients with diabetes were at increased risk of mortality independently of other comorbidities, such as hypertension, chronic obstructive pulmonary disease, heart failure, and chronic renal disease [44]. In a Chinese retrospective cohort study [45], diabetes has been found to be independently related with adverse outcomes in COVID-19, while hypertension only when associated with diabetes was an independent predictor of mortality and ARDS. A report from 1590 COVID-19 patients in China found that diabetes was significantly more prevalent among patients with a worse course of disease than among patients with a less severe form (34.6% vs. 14.3%), being a risk factor for ICU admission and mortality [46]. 

New-onset hyperglycemia or acute decompensated diabetes mellitus have been frequently observed in COVID-19 patients [47,48]. Moreover, susceptibility to other overlapped secondary infections, together with the use of glucocorticoid therapy, can further precipitate acute hyperglycemia [49] with increased plasma osmolality, osmotic polyuria and dehydration, endothelial dysfunction, thrombophilia, and amplified pro-inflammatory cytokine secretion, all key factors in SARS-CoV-2-related multi-organ dysfunction. In addition, binding of SARS-CoV-2 to ACE2 in pancreatic cells can damage islets and reduce insulin release, leading to acute hyperglycemia and transient diabetes [50]. The evidence that diabetes mellitus causes a pro-inflammatory environment has been corroborated by serum levels of inflammation biomarkers, such as interleukin-6 (IL-6), C-reactive protein (CRP), and ferritin, and D-dimer that are markedly higher in COVID-19 patients with diabetes mellitus compared to controls without diabetes [51]. Generally, diabetes mellitus is associated with weakened immune response and enhanced susceptibility to infections, due to inherent neutrophil dysfunction, reduced T-cell responses, and disordered humoral immunity [52]. As other cardio-metabolic comorbidities, type 2 diabetes mellitus bears the fingerprint of RAS imbalance: indeed, chronic activation of “classic RAS” is typical in diabetes and insulin-resistance, despite high sodium intake and high blood pressure, and contributes to microvascular and macrovascular complications and is clearly involved in diabetic kidney disease [53]. Furthermore, chronic hyperglycemia also reduces the ACE2 expression, with a loss of its anti-inflammatory effects and protection of endothelial function, because of a decreased counter-regulation of Ang II [54,55]. 

In the context of ACE/Ang II/AT1R axis hyper-activation, the insulin receptor also uses mitogen-activated protein (MAP) kinase as a downstream mediator of its action [35], mediating growth-factor-like effects, such as vascular smooth muscle growth and cardiac hypertrophy [56]. Even AT1R can activate MAP kinase in its post-receptor cascade, so it can be postulated that these two receptors synergize to exacerbate and perpetuate inflammation, fibrosis, and tissue injury. These findings are in line with the hypothesis that RAS dysregulation could be the backbone in the pathogenesis of severe COVID-19 in patients with diabetes mellitus. This vulnerability may play a synergistic role with the underpinning inflammatory milieu and immune defects associated with diabetes, providing SARS-CoV-2 a pathway for causing prolonged lung injury.

## 5. Arterial Hypertension

Worldwide epidemiologic data provided evidence that hypertension is a pivotal comorbidity related to COVID-19 disease severity. A large meta-analysis [57], including 12 studies on 2389 COVID-19 patients (674 severe cases) found that the severity rate of COVID-19 in hypertensive patients was much higher than in non-hypertensive cases (37.58% vs 19.73%). Moreover, hypertensive patients showed a nearly three-fold higher risk of dying from COVID-19. A community-based observational study [58] examined 1449 hospitalized and non-hospitalized COVID-19 patients in central Massachusetts and found that hypertension was associated with severe outcomes among patients younger than 65 years of age. The results of a meta-analysis, including 60 studies with a total of 51,225 patients hospitalized with COVID-19, are in line with these findings, providing evidence that hypertension was significantly associated with mortality in patients with a mean age < 60 years (OR 3.7) [59]. In another review [60] on 15,794 participants, hypertension has been found to be a significant predictor of ICU admission and mortality. Another retrospective observational study has examined 2877 consecutive patients admitted to Huo Shen Shan Hospital in Wuhan. The authors reported a two-fold increase in the relative risk of mortality in hypertensive individuals compared to non-hypertensive patients. Additionally, hypertensive patients who were not taking anti-hypertensive therapy had a significantly higher risk of mortality than patients on treatment [61]. 

In addition to the hypertensive condition, anti-hypertensive therapy is also likely to affect the course of COVID-19. A large amount of data from both retrospective and prospective studies provided evidence that patients treated with RAS inhibitors [ACE-inhibitors (ACEIs) and angiotensin receptor blockers (ARBs)] tended to have a lower risk of mortality than patients treated with other drugs, disproving initial fears arisen from a possible hyper-production of ACE2 induced by RAS inhibitors, resulting in increased susceptibility to SARS-CoV-2 infection. A large meta-analysis enrolling 101,949 COVID-19 patients found a significant association between treatment with RAS inhibitors and mortality reduction among patients with hypertension [62]. Two nationwide cohort studies, conducted in France and Sweden, reported that taking RAS inhibitors was associated with a lower risk of COVID-19 hospitalization and death [63,64]. An analysis of the HOPE COVID-19 registry revealed that patients receiving RAS inhibitors had lower mortality, ICU admission, and need for mechanical ventilation [65], while an Italian nationwide observational study conducted by the Italian Society of Hypertension found that ACEIs/ARBs did not affect the risk of more severe COVID-19 [66,67]. A French observational study, conducted in a geriatric department, also showed a lower mortality rate in oldest old patients (mean age: 86.3 ± 8.0 years) taking ACEIs/ARBs compared with patients not taking these drug classes [68]. These data on older subjects have been confirmed by other observational studies on hospitalized patients, providing additional evidence on the benefit of RAS inhibitor use in this peculiar population [69,70]. On the other side, ACEIs/ARBs withdrawal was found to be associated with greater risk for complications and mortality in hospitalized COVID-19 patients that were previously taking these drugs, as per indication [71], while several large systematic reviews and cohort studies showed how their assumption/continuation was not harmful, firmly advising against their discontinuation [72,73,74,75]. 

The mechanisms by which hypertension leads to increased risk of worse outcome in COVID-19 are likely to be many. Hypertension is a major cardiovascular risk factor that promotes arteriosclerosis of large and small arteries and accelerates atherosclerosis, leading to cardiovascular disease and death. Left ventricular hypertrophy and myocardial fibrosis, with increased filling pressures and impaired coronary reserve, are key features that contribute to atrial fibrillation, myocardial ischemia, and heart failure with preserved ejection fraction [76]. Certainly, RAS hyper-activation or, at least, “inappropriately normal” renin activity and aldosterone levels are typical of overweight/obese hypertensive patients [33]. In these patients, normal or increased Ang II production results in a stimulation of the ACE/Ang II/AT1R pathway, leading to small arteries constriction, hypertrophy, fibrosis, and tissue injury [77]. It also leads to the activation of NADPH oxidases, with subsequent generation of reactive oxygen species, protein oxidation, and dysregulated cell signaling [78]. Moreover, animal models support a hypothetical link between hypertension and reduced ACE2 expression, corroborated by data showing lower expression of ACE2 mRNA and ACE2 protein expression in the kidneys of hypertensive rats [79]. 

Several findings in animal models [80,81] and humans [82,83] showed how the expression of ACE2 could increase, at least in some organs, after the introduction of ACEIs or ARBs therapy [84]. In any case, ACEIs and ARBs facilitate ACE2 activity with a rebalancing of the “anti-RAS” arm. Indeed, treatment with ARBs can counteract the RAS imbalance through AT1R blockade, while treatment with ACEIs can increase the availability of Ang (1-9) and decrease the degradation of Ang (1-7) [9]. These mechanisms are in agreement with clinical data of a better outcome in COVID-19 patients treated with RAS inhibitors, likely thanks to the rebalancing of the two RAS arms, in addition to the well-known protective effects on the heart and cardiovascular system.

## 6. Dyslipidemia

Another risk factor often associated with overweight/obesity and MetS is dyslipidemia. Although its influence may vary, according to age and the presence of other comorbidities, meta-analyses found that dyslipidemia was associated with higher mortality and disease severity [85,86]. Atherogenic dyslipidemia was more frequent in patients with critical COVID-19 and was significantly associated with intubation and death. High triglycerides levels were associated with high levels of inflammatory biomarkers and poor COVID-19 outcome during hospitalization [87]. A retrospective study also found that decreased serum high-density lipoprotein (HDL) cholesterol levels were associated with COVID-19 severity [88]. Surely, HDL are very complex lipoproteins exerting several functions that go beyond lipid transport and metabolism, and, perhaps, future studies could give a more accurate explanation of these findings.

On the other side, statins, cornerstone drugs in dyslipidemia and cardiovascular disease, demonstrated significant beneficial effects in patients with COVID-19, reducing in-hospital mortality in several observational studies [89,90,91] and meta-analyses [92,93], although not all studies are in agreement [94]. These beneficial properties, already suggested in previous studies on hospitalized patients [69], might be mediated by their speculative pleiotropic effects, including anti-inflammatory, immunomodulatory, and antithrombotic properties, but the atherosclerotic plaques stabilization, thus avoiding acute cardiovascular ischemic events that often complicate severe COVID-19, is likely to play the key role [70,95,96,97,98]. Moreover, several experimental models pointed out their possible inhibitory action on the “classic RAS”, ameliorating Ang II-mediated cardiac hypertrophy and fibrosis [98,99]. In experimental models, statins also promote ACE2 up-regulation via inhibition of the MYD88–NF-κB pro-inflammatory pathway [100]. 

All these data highlight the association between dyslipidemia, the use of lipid-lowering drugs, and COVID-19 severity. Further studies are needed to clarify this linkage, but disposable evidence suggests that dyslipidemia, with its related cardiovascular risk, often reported in obese, diabetic, and hypertensive subjects [101], is a real further risk factor for severe COVID-19 more than a simple biomarker of obesity-related dysmetabolism.

## 7. Male Sex

From the early phases of the pandemic, male sex has been found to be associated to a more severe course of COVID-19 and greater need for intensive care, compared with female sex [102]. Studies found how men had a 59% increased risk for severe outcomes compared to women [103]. Observational studies reported how the vast majority (82%) of patients that needed intensive care were males [104] and how the main determinants of ICU admission were male sex and obesity [105]. In a study on 4062 hospitalized COVID-19 patients in New York City, males had a higher risk of mortality compared to females and were more likely to present with sepsis and hypoxia on admission [106]. A large meta-analysis [107], including 3,111,714 reported global cases of COVID-19, found that males had higher risk of both ICU admission and death compared to females, although no difference has been found in the proportion of males and females infected with SARS-CoV-2. 

Several factors can explain this sex difference. Although the greatest evidence on RAS and sex comes from preclinical studies, both types of sex hormones, estrogens and androgens, likely affect the expression and activity of several RAS components, especially regarding the “classic RAS” pathway, while limited data are available on the interactions with the counter-regulatory RAS components [108]. Estradiol is likely to cause a protective shift in ACE/ACE2 ratio, by both increasing ACE2 and inhibiting ACE expression [109]. Estrogenic activity has been found to inhibit the hemodynamic effects of Ang II and promote the action of ACE2/Ang (1-7) axis in animal models [110]. Estrogen has also been found to decrease tissue AT1R expression and aldosterone production, while testosterone conversely increases ACE activity and tissue AT1R expression [108]. Furthermore, the gene for ACE2 is located in the X chromosome, which could make it susceptible to escaping X-inactivation in women [111]. In an animal model of obesity-associated hypertension, the increase in blood pressure after high-fat diet is attenuated in females compared to males, and this different behavior appears to be mediated by ACE2 activity [112]. In humans, men tend to have higher levels of aldosterone than females, regardless of other confounders, facilitated, at least in part, by higher levels of endogenous Ang II [113]. 

Further studies are needed to disclose all the mechanisms underlying this sex difference, but it appears reasonable that the sex-specific RAS regulation contributes to female protection from severe COVID-19, as well as from some cardio-metabolic conditions found more frequently in men [108,114].

## 8. Older Age

Since the beginning of the pandemic, older age, besides the presence of comorbidities, has been clearly associated with a worse outcome in COVID-19 [67]. In China, data collected by the WHO revealed that the majority of deceased patients with COVID-19 were 70 years or older. Another report from the Chinese Centre for Disease Control and Prevention found that fatality rates were 8% and 15% among people aged 70 to 79 years and 80 years or older, respectively, while case fatality rate among the entire cohort was 2.3% [115]. Palmieri et al. [116] examined the characteristics of 35,595 cases of COVID-19-related deaths in Italy from March 2020 to August 2020, finding that the median age was 80.2, with 57.3% of males; interestingly, they also reported that, in the second phase of the pandemic (June–August 2020), deceased patients with COVID-19 were significantly older (median age 82.8) and with a greater burden of comorbidities. Another Italian study [104] has evaluated a cohort of 3988 critically ill patients with COVID-19 admitted to ICU in Lombardy region, from February 2020 to April 2020; at the multivariate analysis, they found that age over 69 years (hazard ratio 4.25) and male sex (hazard ratio 1.22) were significantly associated with mortality. Furthermore, they also confirmed that hypertension, hypercholesterolemia, heart disease, and diabetes were associated with increased mortality. A large population-based study reported how older age was strongly associated to increased risk of COVID-19-related death: the risk increased with increasing age, up to 20-fold for subjects aged ≥ 80 years compared with subjects aged 50–59 years, independently of comorbidities and other confounders [103]. A post-hoc analysis of the international, multicenter HOPE COVID-19 registry has selected all patients aged ≥ 65 years hospitalized for COVID-19, reporting that patients aged 75 years and older had more in-hospital complications and a significantly higher mortality. Their most prevalent comorbidities were hypertension (69.2%), dyslipidemia (48.6%), heart disease (38.4%), and chronic lung disease (25.3%) [117]. 

Several factors and age-related modifications are responsible for the increased risk of severe COVID-19 and death in older patients: the large spectrum of multiple cardio-metabolic morbidities, leading to a much higher cardiovascular risk, as well as immune-senescence, endothelial dysfunction, limited organ reserve (especially diminished cardiorespiratory function), and other psychosocial and nutritional factors [118]. In this large variety of factors, age-related decline in ACE2 expression, as observed in the lungs of rats [119], may play a non-negligible role. In older people, especially those with cardiovascular comorbidities, reduced ACE2 levels and increased Ang II signaling arrange a pro-inflammatory background. When these subjects are infected with SARS-CoV-2, that leads to a further reduction in ACE2 cell surface expression, there is a consequent overwhelmed amplification of the ACE/Ang II/AT1R pathway that perpetrates microvascular damage and inflammatory effects leading to severe lung injury [120]. It must be recalled that aging has a major role in cardiovascular disease by substantially “giving time” to multiple risk factors, even “borderline” in severity, to produce vascular damage through decades of inappropriate control [121]. When infections strike these older patients, the clinical scenario may often complicate with acute cardiovascular events, leading to more severe clinical course and worse outcome. Furthermore, other conditions often resulting from poor control of cardio-metabolic risk factors, such as chronic kidney disease and vascular dementia, are highly prevalent in older subjects, and are major contributors of severe COVID-19 and death [117]. However, the disequilibrium between ACE and ACE2 activity, a pathophysiological feature of aging, is likely to play a key role in determining the disease severity in older people affected by COVID-19 [67].

## 9. Conclusions

**Figure 2:**
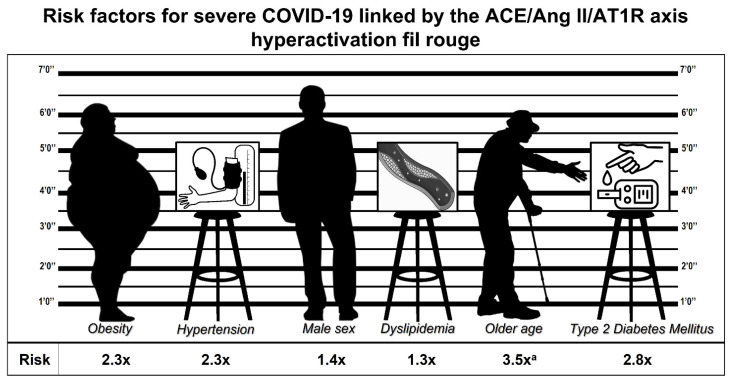
Identikit of the patient at risk for severe COVID-19: “classic RAS” inappropriate activation in a context of lower ACE2 activity as a common feature. Health conditions, cardiometabolic comorbidities, and their assumed relative risk [5,25,43,85,107,117]. a: for patients aged ≥ 75 years.

Titanic efforts have been made, and they are incessantly extended, to understand the pathophysiological mechanisms of COVID-19 in order to prevent its consequences. In this context, our work provides a pathophysiological link that combines several features of patients at higher risk of developing severe complications and dying from SARS-CoV-2 infection, based on RAS dysregulation, which is typical not only of severe COVID-19 but also of the most prevalent cardio-metabolic conditions. Therefore, we can draw a particular phenotype, an “identikit” of the patient characterized by male sex, older age, features of MetS (excessive visceral adiposity with insulin resistance, altered glucose, and lipid metabolism), and arterial hypertension (Figure 2), aiming at detecting patients at higher risk for severe COVID-19 and death. Ad-hoc studies that develop a risk score based on the risk factors taken into account in the present review may allow a rapid identification of these patients, with possible benefits in terms of resource allocation and prognosis. Further studies are needed, to better clarify the pathophysiological bases of COVID-19, in order to arrange more effective instruments of prevention and care that could help us to reduce morbidity and mortality in the long struggle with this tremendous plague. At the moment, the most rational and evidence-based link between the common cardio-metabolic conditions and severe COVID-19 is the one based on the dysregulation of RAS. 

## Data Availability

No new data were created or analyzed in this study. Data sharing is not applicable to this article.
